# Supplementary material for: Maximum Gene-Support Tree
Source: Evol Bioinform Online. 2008 May 15;4:181–91. doi: 10.4137/ebo.s652 (PMC2614190; doi:10.4137/ebo.s652)
Supplement: Table S1 — The names and GenBank GIs of the 36 genes of seven plant species. [file ebo-4-181-s1.doc]

**Table S1.** The names and GenBank GIs of the 36 genes of seven plant species.

| **Serial No** | **Gene name** | ***Arabidopsis thaliana*** | ***Ginkgo biloba*** | ***Oryza sativa*** | ***Picea glauca*** | ***Pinus taeda*** | ***Populus tremula*** | ***Triticum aestivum*** |
| --- | --- | --- | --- | --- | --- | --- | --- | --- |
| 1 | 14-3-3 protein [*Populus x canescens*] | 19867870 | 27918578 | 27576456 | 49056383 | 49011449 | 18007655 | 9358307 |
| 2 | 40S ribosomal protein S24 (RPS24A) [*Arabidopsis thaliana*] | 8333422 | 27889350 | 3767644 | 40775252 | 5903897 | 3853886 | 38999197 |
| 3 | 40S ribosomal protein S5 (RPS5A) [*Arabidopsis thaliana*] | 5841333 | 27888835 | 2800554 | 50174597 | 49011331 | 24005088 | 25198336 |
| 4 | 60S ribosomal protein L11 (RPL11B) [*Arabidopsis thaliana*] | 37427419 | 27888766 | 25803988 | 40775248 | 34351444 | 23996035 | 32656103 |
| 5 | 60S ribosomal protein L13E [Picea abies] | 16605 | 27888733 | 2311423 | 50150835 | 37566969 | 23995674 | 25222054 |
| 6 | 60S ribosomal protein L30 | 19866717 | 27889091 | 2311814 | 49053100 | 8173511 | 23961836 | 25196338 |
| 7 | Actin-depolymerizing factor, putative [*Arabidopsis thaliana*] | 1269231 | 27888971 | 7212631 | 40767649 | 3366019 | 3853246 | 25557489 |
| 8 | AT4g27960 *[Arabidopsis thaliana*] | 8333427 | 27888932 | 14713790 | 50152668 | 34355474 | 24019749 | 11117148 |
| 9 | Calmodulin [*Arachis hypogaea*] | 47829277 | 27888469 | 47506412 | 40760719 | 34354209 | 3856858 | 9739999 |
| 10 | Chlorophyll a/b binding protein CP29 [*Vigna radiata*] | 32885927 | 27888931 | 29684585 | 50178477 | 48943124 | 24005334 | 8845049 |
| 11 | Chlorophyll a/b-binding protein [*Picea glauca*] | 315905 | 27918813 | 4716678 | 50175858 | 47579825 | 14491444 | 21838448 |
| 12 | Chlorophyll a-b binding protein 36, chloroplast precursor (LHCII type I CAB-36) (LHCP) | 47829474 | 27919128 | 36355271 | 50175858 | 47578726 | 24019830 | 32784307 |
| 13 | Defender against cell death (DAD-1) | 19864830 | 27888478 | 427990 | 50176691 | 51498938 | 24003441 | 9741968 |
| 14 | Disease-resistent-related protein [*Oryza sativa*] | 47828161 | 27888660 | 700897 | 49051093 | 5043937 | 24006336 | 9696341 |
| 15 | Histone H2B [*Arabidopsis thaliana*] | 47828295 | 27918825 | 25800711 | 40773517 | 34490270 | 3855806 | 9412282 |
| 16 | Hypothetical protein F11P17.12 [imported] [*Arabidopsis thaliana*] | 8722938 | 27919094 | 29684937 | 40773347 | 49009735 | 24020750 | 20442002 |
| 17 | Light-harvesting chlorophyll a/b binding protein of photosystem II [*Pseudotsuga menziesii*] | 8679534 | 27888619 | 29612907 | 50155019 | 48944321 | 38573820 | 25444513 |
| 18 | OSJNBb0012E24.5 [Oryza sativa (japonica cultivar-group)] | 48977972 | 27888971 | 7212631 | 50160305 | 20665642 | 18006760 | 9698530 |
| 19 | Plastid developmental protein DAG, putative [*Arabidopsis thaliana*] | 19875687 | 27888899 | 29657005 | 50165257 | 34341971 | 14491897 | 22029060 |
| 20 | Polyubiquitin [*Fragaria x ananassa*] | 47830375 | 27888607 | 29661417 | 49126509 | 34353685 | 38594187 | 38989668 |
| 21 | Polyubiquitin 6 – rice | 23302939 | 27888607 | 29678711 | 49049866 | 34349177 | 38594168 | 21319828 |
| 22 | Probable phospholipid hydroperoxide glutathione peroxidase (PHGPx) | 8334424 | 27888930 | 11442957 | 50150052 | 51495452 | 23997414 | 25202466 |
| 23 | Putative chlorophyll A-B binding protein type I [*Pinus pinaster*] | 8679534 | 27888619 | 14981614 | 49049718 | 48944321 | 38573820 | 25444513 |
| 24 | Putative ethylene-responsive transcriptional coactivator [*Oryza sativa* (japonica cultivar-group)] | 47830185 | 27889065 | 51529706 | 49125634 | 34505949 | 18014373 | 51529706 |
| 25 | Putative histone H2A [*Pinus pinaster*] | 48976448 | 27888779 | 3767070 | 40777088 | 48941761 | 24076130 | 9421764 |
| 26 | Putative nifU-like protein [*Oryza sativa* (japonica cultivar-group)] | 47828968 | 27918576 | 25798405 | 50145201 | 34360268 | 24109343 | 9696756 |
| 27 | Putative ubiquitin [Pinus pinaster] | 937826 | 27888783 | 33659175 | 40774023 | 21787910 | 18006929 | 24980594 |
| 28 | Ribosomal protein S11 (probable start codon at bp 67) | 47828662 | 27888499 | 5607432 | 50149659 | 34347714 | 24004330 | 20081851 |
| 29 | Ribulose bisphosphate carboxylase small chain, chloroplast precursor (RuBisCO small subunit) | 48976218 | 27918803 | 15855145 | 49052023 | 48943929 | 14488877 | 24978689 |
| 30 | Similar to CG31613-PA [*Rattus norvegicus*] | 19874758 | 27888660 | 33664790 | 40774831 | 34361051 | 23997296 | 20088147 |
| 31 | Thioredoxin M-type, chloroplast precursor (TRX-M) | 47829038 | 27889121 | 6528815 | 50171743 | 48942681 | 23996233 | 25429750 |
| 32 | Tubulin alpha-1 chain | 8680218 | 27919054 | 29661051 | 49135517 | 34349366 | 38571735 | 39570710 |
| 33 | Type I (26 kD) CP29 polypeptide [*Lycopersicon esculentum*] | 8679534 | 27889103 | 14980886 | 49049804 | 48943221 | 38574381 | 12865470 |
| 34 | Ubiquitin homolog [Arabidopsis thaliana] | 19800556 | 27919349 | 29661418 | 49062199 | 49010877 | 38572251 | 23070605 |
| 35 | Ubiquitin-conjugating enzyme UBC2 [*Mesembryanthemum crystallinum*] | 2747787 | 27888783 | 27577233 | 40774696 | 3365470 | 14490645 | 9365286 |
| 36 | Xyloglucan endo-1,4-beta-D-glucanase (EC 3.2.1.-) 1 - common nasturtium | 47831742 | 27888476 | 25797725 | 40780279 | 34506595 | 3855398 | 9360221 |
